# Post-Translational Modifications of STING: A Potential Therapeutic Target

**DOI:** 10.3389/fimmu.2022.888147

**Published:** 2022-05-06

**Authors:** Jiaqi Kang, Jie Wu, Qinjie Liu, Xiuwen Wu, Yun Zhao, Jianan Ren

**Affiliations:** ^1^ Research Institute of General Surgery, Affiliated Jinling Hospital, Medical School of Nanjing University, Nanjing, China; ^2^ Department of General Surgery, BenQ Medical Center, The Affiliated BenQ Hospital of Nanjing Medical University, Nanjing, China

**Keywords:** STING, post translational modification, targeted therapy, diseases, phosphorylation

## Abstract

Stimulator of interferon genes (STING) is an endoplasmic-reticulum resident protein, playing essential roles in immune responses against microbial infections. However, over-activation of STING is accompanied by excessive inflammation and results in various diseases, including autoinflammatory diseases and cancers. Therefore, precise regulation of STING activities is critical for adequate immune protection while limiting abnormal tissue damage. Numerous mechanisms regulate STING to maintain homeostasis, including protein-protein interaction and molecular modification. Among these, post-translational modifications (PTMs) are key to accurately orchestrating the activation and degradation of STING by temporarily changing the structure of STING. In this review, we focus on the emerging roles of PTMs that regulate activation and inhibition of STING, and provide insights into the roles of the PTMs of STING in disease pathogenesis and as potential targeted therapy.

## Introduction

Innate immunity is the front line of defense, protecting the host from microbial invasion and triggering adaptive immunity to eradicate infections. When pathogen-associated molecular patterns (PAMPs), including bacterial lipopolysaccharide (LPS) and viral nucleic acids, invade the body with or without tissue damage, pattern recognition receptors (PRRs), which are located on the cell membrane or in the cytoplasm, can be activated and mediate inflammatory and antiviral pathways to deal with infection (1, 2). In addition, tissue and cell damage are accompanied by damage-associated molecular patterns (DAMPs), such as abnormal DNA and cell organelle fragments, which can also be recognized by PRRs and induce cascades of inflammatory signaling pathways (2). PRRs are protein family including Toll-like receptors (TLRs), C-type lectin receptors (CLRs), NOD-like receptors (NLRs), retinoic acid-inducible gene-I (RIG-I)-like receptors (RLRs), cyclic GMP-AMP synthase (cGAS), IFN-γ-inducible protein 16 (IFI16), absent in melanoma 2 (AIM2) and multiple DNA sensors (1–3). Different PRRs can be activated by different PAMPs, which then trigger innate immune responses to combat infection (1–4).

Specifically, cGAS, a protein that can enter and exit the nucleus, is recruited to abnormal DNA, which is classified into exogenous pathogenic DNA or endogenous instable heterotopic DNA, leading to a conformational change to bind to DNA (5–7). After that, AMP and GMP are catalyzed by cGAS, releasing the second messenger 2’3’-cyclic GMP-AMP (cGAMP), which functions as the second messenger to initiate adapter protein stimulator of interferon genes (STING) on the endoplasmic reticulum (ER) membrane (7, 8). STING then relocates to the Golgi apparatus, leading to production of cytokines, including type I interferon and other cytokines (7–10). Different immune cells can also produce various cytokines through cGAS- STING pathway to perform their respective function (11).

To date, STING has been generally considered to play a necessary role in immunity and inflammation. However, the regulatory mechanism of STING remains unclear. In addition to the direct interaction between cyclic dinucleotides and STING, post-translation modifications (PTMs) and protein-protein interactions could also alter STING function (4, 9, 12, 13). PTMs of proteins have been recognized as an important regulatory switch to temporally change the functions of proteins in cells. Most cellular proteins can be decorated by a diverse range of PTMs, including phosphorylation, acetylation, methylation, glutamylation, ADP-ribosylation, SUMOylation, and ubiquitination (14–17). In this review, we review the PTMs and function of STING signaling, highlighting the potential targeted therapy afforded by PTMs of STING.

## An Overview of STING Pathway

STING, namely stimulator of interferon genes, also known as TMEM173, MITA, ERIS, and MPYS (18, 19), is a ~40-kDa trans-membrane protein located on the ER membrane (20, 21). It is composed of a short N-terminal cytosolic segment, four trans-membranes (TM) located in the ER membrane, a cytosolic ligand-binding domain (LBD), and a C-terminal tail (CTT) which is responsible for binding TBK1 (19, 21). It exists widely in nature, not only humans, but also chicken (22), shrimp (23), bacteria (24), and *Drosophila (* 25), functioning as pathogen sensors to avoid infection.

As previously mentioned, cGAMP, catalyzed by cGAS, can alter the conformation of STING, resulting in the inward rotation of the two wings of STING toward each other. This process leads to closure of the ligand binding pocket and activation of STING (7). Then STING traffics in the form of COP-II vesicles from ER to ER-Golgi intermediate compartments (ERGIC) (26, 27). Some vesicles loaded with STING serve as a membrane source for modification by the ubiquitin-like protein LC3, which is a key step in autophagosome biogenesis (26). Most STING-coated ERGIC vesicles continue to traffic through the Golgi and post-Golgi endosomes. On the ERGIC membrane, STING recruits TBK1(tank binding kinase 1) and IKK (inhibitor of kappa B kinase), then TBK1 auto-phosphorylates and STING, IRF3 and IκBα are phosphorylated (7, 27). Phosphorylated IRF3 dimerizes and translocates to activate transcription of type I IFN and interferon-stimulated genes (ISGs). Phosphorylation of IκBα results in translocation of NF-κB to the nucleus, leading to transcription of genes encoding pro-inflammatory cytokines and chemokines such as IL-6 and TNF (28).

In addition, micronuclei (29), mtDNA (30), abnormal cell cycle (31), and cytoplasmic chromatin fragments (32) can activate STING through cGAS- dependent way. Several stimuli other than cGAMP, which is catalyzed by cGAS, for example, bacterial or virus cyclic dinucleotides (CDNs) (33, 34), can also activate STING directly. Apart from the production of type I interferon and cytokines, STING can also be associated with other biological and pathological process, such as ER stress (35), oxidative stress (36), fatty acid metabolism (37), Ca^2+^ homeostasis (38, 39), T cell proliferation (40), senescence (32) and so on. Moreover, STING can also participate in cell death pathways (41), including autophagy (30, 42), apoptosis (43), pyroptosis (44, 45), ferroptosis (46), necroptosis (47, 48), mitotic death (49), immunogenic cell death(ICD) (50). However, sometimes STING can help herpes virus assemble viral genome to host cell’s nucleus and survive in host cells, leading to severer virus infection at the early stage of infection (51).

Now that STING plays critical roles in biochemical processes, the expression and function of STING are tightly regulated. Apart from direct interaction between cGAMP and STING, protein-protein interaction and post-translational modification (PTMs) are also important (9, 12). Hundreds of proteins may interact with STING and affect its function (52). However, the regulation of STING in cells are basically dependent on the PTMs. In a word, PTMs are the key to regulating protein function and play a critical role in modulation of STING. Thus, we review PTMs of STING and prospect related targeted therapy.

## Modification and Regulation of STING

**Figure 1:**
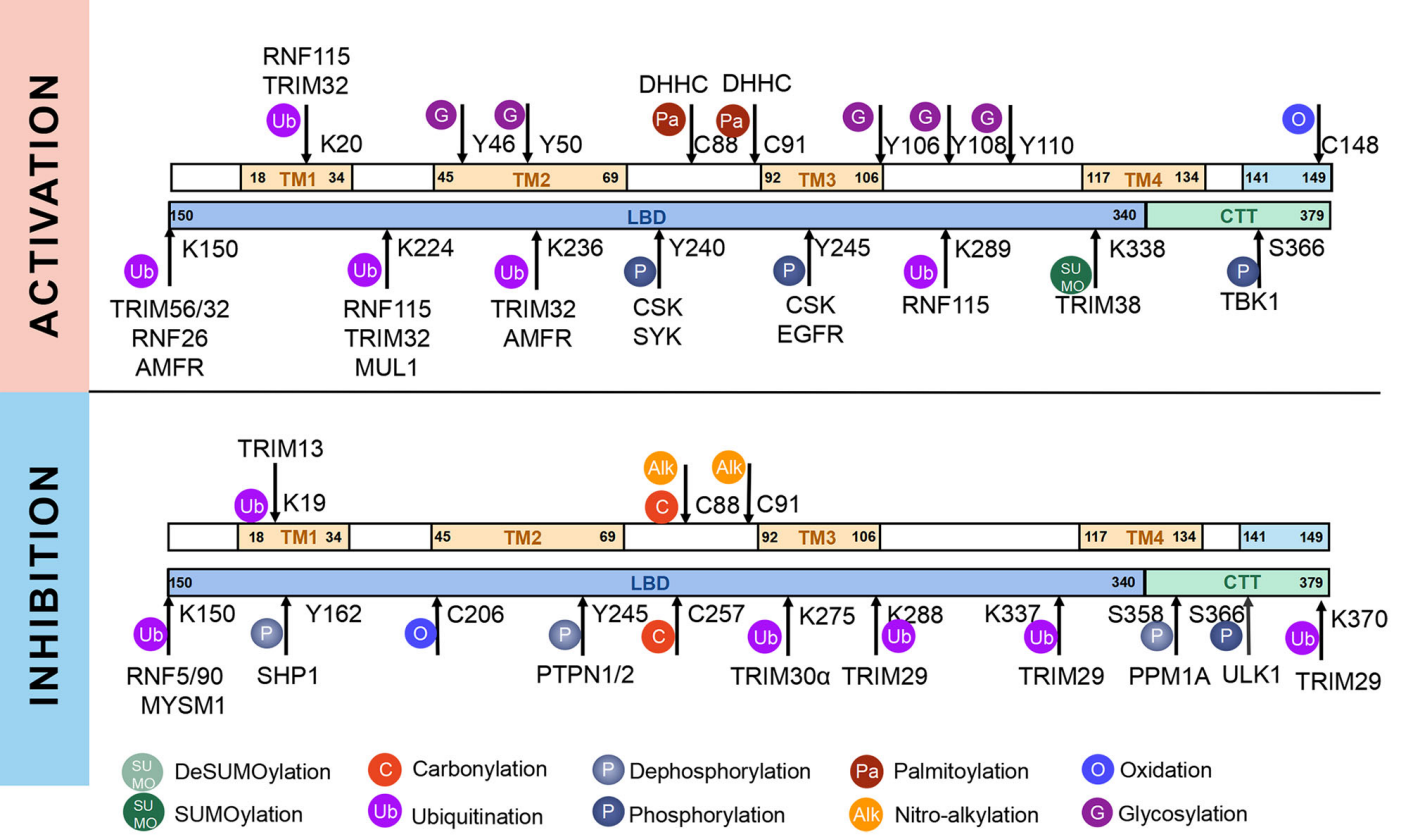
Post-translational modifications of STING. STING can be modified by phosphorylation and dephosphorylation, ubiquitination and deubiquitination, SUMOylation and deSUMOylation, palmitoylation, alkylation, glycosylation, carbonylation, oxidation, and disulfide bond, which are indicated according to the keys in the bottom. The PTMs that promote activation of STING are shown above, whereas those that inhibit STING activation are shown below. Related enzymes are presented around the PTMs.

**Figure 2:**
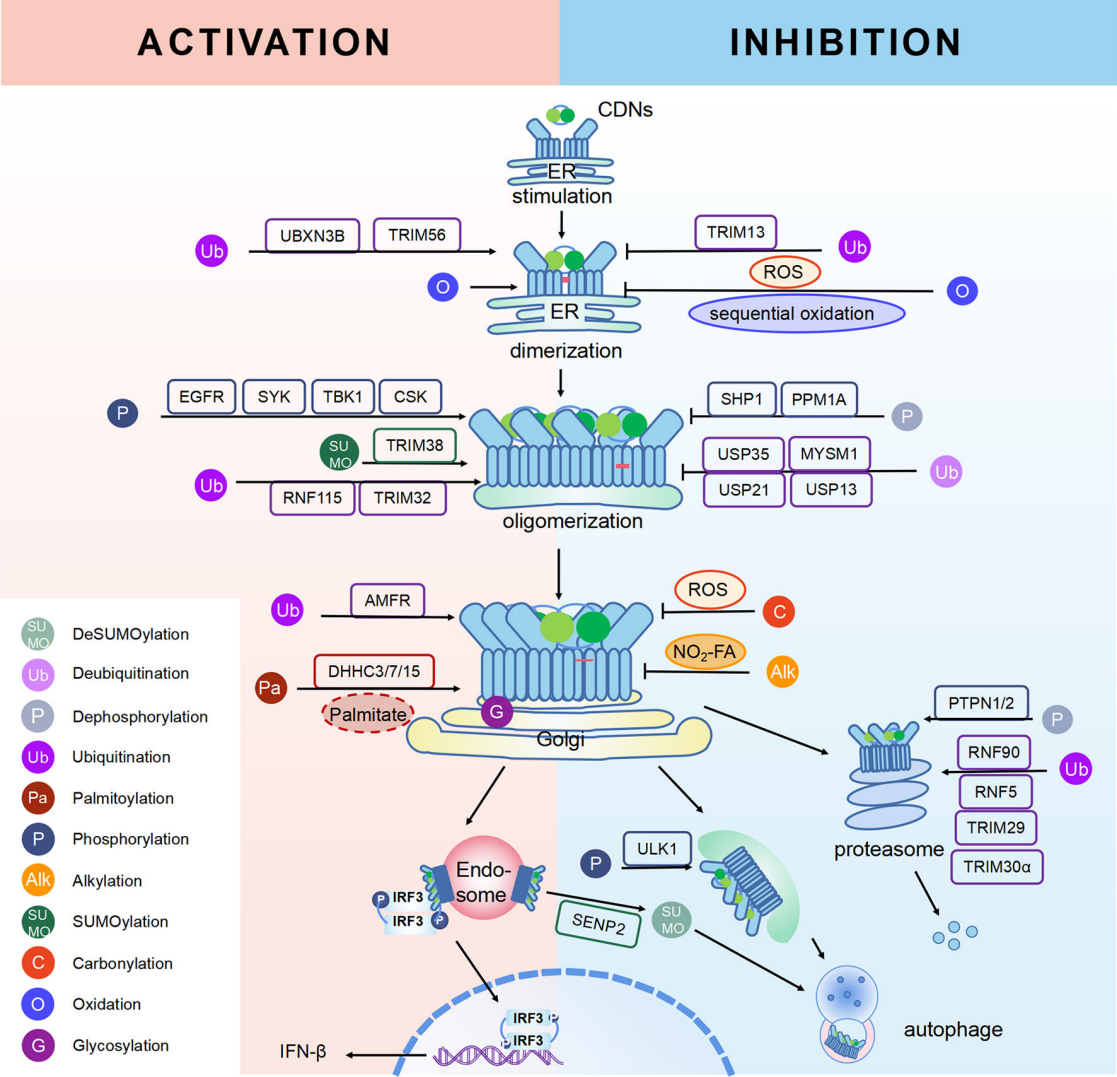
PTMs in STING pathway. PTMs are going along with the life of STING, from activation to degradation. When CDNs bind to STING, STING is recruited and dimerized with the help of reversible oxidation and disulfide bond. SUMOylation and ubiquitination can help STING with oligomerization, while phosphorylation is related to the activation of STING. Then STING transports from ER to Golgi. Palmitoylation and sGAGs could help STING reside on Golgi, thus continuous activation of STING leads to increasing pro-inflammatory cytokines. On the other hand, inhibition of STING happens along with activation to achieve cellular homeostasis. Sequential oxidation inhibits STING dimerization. Dephosphorylation and deubiquitination of STING decrease the oligomerization of STING. Also, carbonylation caused by ROS and alkylation caused by NO_2_-FAs can competitively inhibit palmitoylation and block sequential residence of STING on Golgi, thus immune responses are inhibited. In addition, dephosphorylation, ubiquitination and deSUMOylation could facilitate protein degradation to maintain proteostasis.

STING, a protein located in the ER, is composed of 379aa in human. Some residues of STING can be modified for its compartmentalization, dimerization, oligomerization, trafficking and degradation, which regulate immunological and other processes (**Figure 1**, **2**). The consequence of PTMs is dependent on amino acid residue, type of modification group, thus biochemical process can be fully developed in cells. Therefore, we focus on different PTMs of STING and propose critical role of PTMs in activation or inhibition of STING. Modified residues and related proteins are shown in **Figure 1**. While **Figure 2** shows how PTMs of STING affects STING pathway. Therefore, some PTMs in **Figure 1** are not shown in **Figure 2** due to the lack of literature.

### Phosphorylation and Dephosphorylation

Phosphorylation is the most common PTMs in mammalian cells. It means the addition of phosphate groups to proteins, especially to amino acid residues, such as Ser and Thr (53). The addition or removal of phosphate groups (dephosphorylation) acts as a biological “on/off” in many reactions, including modulation of STING (9) (**Figure 2**).

It is confirmed that, oligomerized STING recruited TBK1, then TBK1 phosphorylated S366 of human STING (S365 in mice), with the consequence of classic activation of STING pathway (54–56). CSK (C-terminal src kinase), another tyrosine kinase expressed broadly among mammalian cells, phosphorylates STING at Y240 and Y245 and activates immune responses *via* promoting aggregation of STING after HSV-1 infection (57). PPP6C, namely protein phosphatase 6 catalytic subunit, is a phosphatase. Knockdown of PPP6C greatly increased STING phosphorylation at S366 after dsDNA stimulation to enhance immune response (58). Y245 of STING can also be phosphorylated directly by EGFR (epidermal growth factor receptor) for STING trafficking to endosomes, thus type I-IFN are produced (59). SYK (spleen tyrosine kinase), another kinase, could also phosphorylate Y240 of STING and promote activation of STING (60).

However, to achieve cellular homeostasis, STING pathway can also be down-regulated *via* phosphorylation and dephosphorylation (**Figure 2**). For instance, STING is subsequently phosphorylated by UNC-51-like kinase (ULK1) at S366 so that sustained innate immune responses can be prevented (61). Dephosphorylation of S358 by Mg2+/Mn2+-dependent protein phosphatase 1A (PPM1A) can also inhibit STING aggregation and STING-dependent pathway (62). SHP1(SH2-containing protein tyrosine phosphatase), a phosphatase, can dephosphorylate STING at Y162, blocking the K63-linked ubiquitination of STING at K337 and inhibiting STING pathway (63). Tyrosine-protein phosphatase nonreceptor type (PTPN) 1 and 2 can dephosphorylate STING at Y245 with the consequence of degradation of STING *via* the ubiquitin-independent proteasomal pathway (64). In a word, phosphorylation and dephosphorylation of STING play important roles in modulation of STING. The imbalance between phosphorylation and dephosphorylation could be critical mechanism in development of diseases.

### Ubiquitylation and Deubiquitylation

Ubiquitylation, a highly-conserved modification of protein, is the second most common PTM for proteins, after only phosphorylation (53). It can be divided into three types due to structural characteristics: mono-ubiquitination, poly-ubiquitination and branched ubiquitination (65). Ubiquitylation (Ub) is initiated by a cascade of enzymatic reactions, which is catalyzed by Ub-activating (E1), Ub-conjugating (E2) and Ub-ligating (E3) enzymes (65). Firstly, ubiquitin (Ub) is activated by E1 in an ATP-dependent manner and then is transferred to E2. Then, specific E3 catalyzes the transfer of Ub from E2 to a specific substrate protein with the assistance of E1 (66). As a result, Ub is assembled covalently to the specific protein, especially to lysine residues, resulting in the regulation of quality and quantity of proteins through degradation in various physiological and/or pathological conditions. Deubiquitylation happens along with ubiquitylation, and both of them together affect the maturation and degradation of proteins.

Specifically, STING can also be modified by Ub *via* E3 ubiquitin ligase (**Figure 2**). RNF115(RING finger protein) can catalyze K63-linked polyubiquitination of STING at K224/20/289, promoting the translocation of STING from ER to Golgi (67). TRIM56(tripartite motif protein) induces K63 linkage ubiquitination of STING at K150 due to stimulation of exogenous DNA, and promotes STING dimerization and recruitment of TBK1 (68). UBXN3B (ubiquitin regulatory X domain-containing proteins 3 b) facilitates TRIM56-dependent K63-linked ubiquitination of STING, thus leading to dimerization, trafficking, and activation STING signaling (69). MUL1 (mitochondrial E3 ubiquitin protein ligase 1) can catalyze K63-linked polyubiquitination of STING at K224 to help activation of STING (70). TRIM32 targeted STING for K63-linked ubiquitination at K20/150/224/236 and promoted the STING pathway (71). However, HSV1 VP1/2 can deubiquitinate K63-linked polyubiquitination of STING, which is mediated by TRIM32, thus help HSV escape from immune responses and promote brain infections (72). Reverse transcriptase domain of HBV can also physically bind to STING and significantly reduces the K63-linked polyubiquitination of STING, protecting HBV from innate immune responses (73). These indicate that pathogen has developed PTMs targeted strategy to evade from host immune system. Autocrine motility factor receptor (AMFR) catalyzed the K27-linked polyubiquitination of STING in an insulin-induced gene 1 (INSIG1)-dependent manner, recruiting TANK-binding kinase 1 (TBK1) and facilitating its translocation to the perinuclear microsomes (74). Therefore, K63-linked and K-27 linked polyubiquitination play important roles in activation of STING pathway.

While RNF5 and RNF90 target STING at K150 for K48-linked ubiquitination and lead to degradation of STING after viral infection (75, 76). However, RNF26 promotes K11-linked polyubiquitination of STING at K150 and protects STING from RNF5-mediated K48-linked polyubiquitination and degradation, thus enhancing quick and efficient anti-viral responses (77).TRIM29 catalyzes K48-linked polyubiquitination at K288/337/370 and promotes the degradation of STING (78, 79). TRIM30α induces K48-linked ubiquitination of STING at K275 for proteasome-dependent degradation (80). There is another interesting protein, death-associated protein kinase 3(DAPK3), which modifies STING ubiquitination differently in different situations. When cells are in basic condition, DAPK3 inhibited STING K48-linked poly-ubiquitination and proteasome-mediated degradation. By contrast, DAPK3 is required for STING K63-linked poly-ubiquitination and STING-TBK1 interaction after cGAMP stimulation (81). Recently, TRIM13 is discovered to bind to STING at resting state. After HSV-1 infection, K6-linked polyubiquitination at K19 is triggered by TRIM13, thus promoting degradation of STING (82). In a word, different types of ubiquitination precisely regulate activation or degradation of STING, including K11-linked, K27-linked, K48-linked, K63-linked, K6-linked, leading to different effects in immune responses (69). K27-linked, K11-linked and K63-linked polyubiquitination can promote transport and activation of STING, while K6-linked, and K48-linked polyubiquitination mostly lead to degradation of STING (**Figures 1**, **2**).

On the other hand, ubiquitin-specific protease (USP)21, a deubiquitinating enzyme, hydrolyzes K27/63-linked polyubiquitin chain on STING, resulting in negative regulation of STING and significant decrease of type I interferons (83). USP13 uncouples K27-linked polyubiquitin chains from STING and prevents the recruitment of TBK1 to inhibit STING pathway (84). USP35 directly removes K11-, K27-, K63-linked polyubiquitin of STING, thus inhibits phosphorylation and multimerization of STING to limit STING signaling (85). The MYSM1 (Myb-like, SWIRM, and MPN domains 1 protein), interacts with STING and cleaves STING K63-linked ubiquitination at K150 to inhibit cGAS-STING signaling (86). On the other hand, OTUD5 and USP20 catalyze the K48-linked deubiquitination of STING and inhibit STING degradation, thus maintain the stability of STING and promote activation of STING pathway (87–89). In conclusion, targeted ubiquitination could be potential therapeutic approach (**Figures 1**, **2**).

### SUMOylation and deSUMOylation

Small ubiquitin-related modifier (SUMO) is a widely expressed Ub-like protein, which is similar with Ub in structure and enzymatic cascade (16). There are three major SUMOs. SUMO-1 usually modifies a substrate as a monomer; while SUMO-2/3 can form poly-SUMO chains. Both SUMO-1 and poly-SUMO chains can interact with other proteins through SUMO-interactive motif (SIM) (16). Thus, SUMO modification participated mainly in enhancing protein-protein interaction and regulating proteins’ localization, stability and activity (16, 65). As for STING, TRIM38 mediates the SUMOylation of STING at K338 to inhibit STING degradation, promoting oligomerization of STING and recruitment of IRF3 (90). On the other hand, at the late stage of infection, STING is deSUMOylated by SUMO-specific protease (SENP)2 after phosphorylation at S366, eventually leading to STING degradation and dampening innate immune responses (90)(**Figures 1**, **2**). More studies are needed to figure out whether other SUMO-enzymes play roles in regulation of STING, for example, SAE1 (SUMO-activating enzyme subunit 1), a subunit of SUMO-activating enzyme, which might interact with STING and plays a role in regulation of STING (52).

### Palmitoylation

Palmitoylation, or S-palmitoylation, a type of lipidation, can make proteins bind non-polar structures more tightly, with important function for the localization, diffusion, and physical interactions of these proteins within the cell. It is namely related to palmitic acid (PA), which comes out either with intrinsic fatty acid synthesis, or fatty acid uptake from outside of cells (91). However, studies have also discovered that palmitoylation is not only associated with PA concentration, but also with the zinc finger DHHC-type containing (ZDHHC) family of palmitoyl S-acyltransferases (PATs), ZDHHC3, ZDHHC7, ZDHHC15, which are mostly localized to the ER and Golgi apparatus (92, 93). On the other hand, depalmitoylation, which means the removal of S-palmitoylation, can be catalyzed by some depalmitoylases, which belong to the serine hydrolase family, including APT1 (LYPLA1), APT2 (LYPLA2) and so on (94).

It has been confirmed that the palmitoylation of cysteines 88 and 91 of STING participates in regulation of STING, but not trafficking of STING (95)(**Figures 1**, **2**). Palmitoylation of STING triggers composition of a multimeric complex at the lipid rafts of the *trans*-Golgi network, which triggers STING interacting with TBK1 (96). Suppression of palmitoylation with 2-bromopalmitate (2-BP) and hydoxylamine eliminates the transcription of downstream inflammatory cytokine genes, thus the phenotype of STING-associated vasculopathy with onset in infancy (SAVI, an auto-inflammatory disease related to gain-of-function mutations of STING) could be improved. Mutation of C88/91 inhibits palmitoylation and decreases the activation of STING-dependent host defense genes (95). However, it is still unknown whether STING can be depalmitoylated by any of the depalmitoylases. More studies are needed to investigate functions of depalmitoylated STING and related mechanisms.

### Nitro-Alkylation

Nitro-alkylation, which is related to nitro-fatty acids (NO_2_-FAs), has been discovered as potent inhibitors of STING signaling. NO_2_-FAs is a recently discovered group of bioactive lipids with anti-inflammatory and tissue protective functions (97). It is produced mainly in gastrointestinal tract during digestion. Meanwhile, it can come out locally to modify specific proteins through Michael addition reactions, so that inflammation responses can be regulated accurately (98).

As mentioned above, there are two cysteine residues in the N-terminal region of STING, Cys88/91, which can be recognized by NO_2_-FAs to covalently interact with, resulting in nitro-alkylation of STING (92). Once STING is nitro-alkylated by NO_2_-FAs, palmitoylation of STING is abolished and STING pathway is inhibited (92). In addition, treatment with nitro-fatty acids is sufficient to inhibit production of type I IFN in fibroblasts derived from SAVI patients (99). In conclusion, nitro-alkylation can be a potent inhibitor of palmitoylation in cells and disease (**Figures 1**, **2**). However, there is still no enzymes covered which is responsible for alkylation of STING, or other proteins. In addition, studies have suggested that, prostaglandin reductase-1 (PtGR-1) promotes nitroalkene transition to inactive nitroalkanes, thus decreases alkylation of protein (100). However, it is still not clear, whether PtGR-1 affects regulation of STING, and how palmitoylation could be replaced or inhibited by alkylation under physiological conditions. More studies are needed to figure out deeper mechanisms.

### Glycosylation

Glycosylation is one of the most diverse post-translational modifications in eukaryotic cells (101). Proteins are glycosylated by either enzymes or interaction directly with glucose (aldehyde form) through lysine and arginine residues in proteins, and eventually leading to advanced glycation end products so that participating in biological or pathological process (101). Glycosylation of proteins are complex, indicated by the diverse types of glycans, the multiple positions of glycans, various structures of glycoproteins and different glycosyl-transferase enzymes, leading to various functions of proteins (102). Studies have discovered that, N41 of STING in mice could be a potential N-linked glycosylation site, however, no N-linked glycosylation was detected with deglycosylation experiments (43). Another experiment showed that, there are four putative N-glycosylation sites of STING in *Drosophila*, N84, N187, N270, N333. Among two forms of STING in drosophila (long form and short form), only long form of STING could be glycosylated thus promoting residence of STING on ER (103). However, the glycosylation of STING in human has not been studied well for unknown reasons.

Glycosaminoglycans (GAGs) are linear acidic polysaccharides, which can be divided into many groups (104). GAGs are subsequently modified by epimerization and sulfation to produce sulfated GAGs (sGAGs) (105). Researchers have discovered that sGAGs can interact with various proteins through their negatively charged sulfate groups (106). Studies have suggested that STING translocates to sGAG-containing vesicles after vaccinia virus infection and evolutionally-conserved bounds to sGAGs through its luminal, positively charged, and polar residues (**Figures 1**, **2**). It is hypothesized that STING palmitoylation facilitates its clustering into lipid rafts on the Golgi apparatus from the STING cytosolic side, while sGAGs induce STING polymerization from the STING luminal side, and both of two methods together lead to full activation of STING and TBK1 (107). However, whether STING is glycosylated and regulated by other substances, especially glucose, are still unclear.

### Carbonylation

Carbonylation, one of the most harmful irreversible oxidative protein modifications, is linked to lipid peroxidation. It is considered as a major hallmark of oxidative stress-related disorders, leading to biomolecule malfunctions and eventually cell death (108, 109). A large amount of evidence has indicated the role of carbonylated proteins in the initiation of inflammation and autoimmune responses (110). However, situation becomes different in STING. There are two carbonylation sites of STING, namely residues C88 and C257, which are conserved across species (111). It is confirmed that both HSV-1 infection and 4-hydroxynonenal (4-HNE, a type of lipid peroxidation metabolite) induce STING carbonylation through lipid peroxidation, preventing the palmitoylation and translocation of STING from the ER to the Golgi, with the consequence of down-regulating immune responses (**Figure 2**). While glutathione peroxidase 4 (GPX4) inhibits STING carbonylation and promote activation of STING. Although the critical carbonylation site of STING has been discovered, there are still many questions to be resolved: why does carbonylation of STING result in inhibition of inflammation, which is different from that of other proteins? Is there a deeper mechanism regulating inflammatory phenotype after carbonylation of proteins? Also, there are still doubts whether carbonylation could be a therapeutic target.

### Reversible Oxidation and Others

Reversible oxidation is a type of reversible oxidative PTM, which is generally related to oxidation of cysteine (Cys), namely Cys ox-PTMs (112). Reversible Cys ox-PTMs consist of various patterns, including S-sulfenylation (Cys-SOH), S-glutathionylation (Cys-SSG), and disulfide bond (113). The characteristics of this oxidation pattern are fast and reversible, contributing to quick transition of protein function, so that cells can adapt to a complicated environment. Studies have discovered that the reversible oxidation of C148 and C206 of STING in cells participate in the contradictory regulation of STING. C148 of STING is oxidized independent on CDNs interaction in basic state. And this oxidation state of C148 makes for the binding of 2’3’-cGAMP to STING (114). However, sequential oxidation of C206 of STING in response to 2’3’-cGAMP leads to a conformational change which inhibits the phosphorylation of S366 and prevents over-activation of STING (114). In addition, excessive ROS induced by viral infection can oxidize C148 and inhibit polymerization and activation of STING, thus helping virus evade from cellular defenses (115). However, it is still unclear, whether other Cys of STING can be oxidized and play roles in regulation of STING. More researches are needed (**Figures 1**, **2**).

It is suggested that disulfide bond is also a type of reversible oxidation, which mainly exists in secreted proteins. Disulfide bond is formed by Cys in the oxidizing environment of the cytosol and in the luminal part of proteins (lumen of mitochondria, ER, etc), with the consequence of a more stable structure of protein. There are five cysteines in the cytosolic domain of STING, only one of which (C148) are engaged in disulfide bonds (116). It is discovered that 2’,3’-cGAMP induces closing of the human STING homo-dimer and leads to the formation of disulfide bond *via* C148. When C148 is mutated to alanine, the affinity of STING to cGAMP is weaker (116). However, more studies are needed to figure out, why disulfide bonds of STING can be produced in the cytosol, and whether disulfide bonds are associated with phase separation. Due to the important role of disulfide bond, it could be potential therapeutic target to improve STING-related auto-inflammatory diseases (**Figures 1**, **2**).

There are still other PTMs which are important in biological and pathological processes, such as acetylation and deacetylation, methylation, biotinylation, ribosylation, carboxylation. However, there is still no evidence favoring these PTMs on STING. Therefore, more research and efforts should be taken into to discover more detailed regulatory mechanisms of STING. Understanding and exploring the underlying network of PTMs may provide new idea of targeted STING therapy.

## PTM Related Diseases and Targeted Therapy

**Figure 3:**
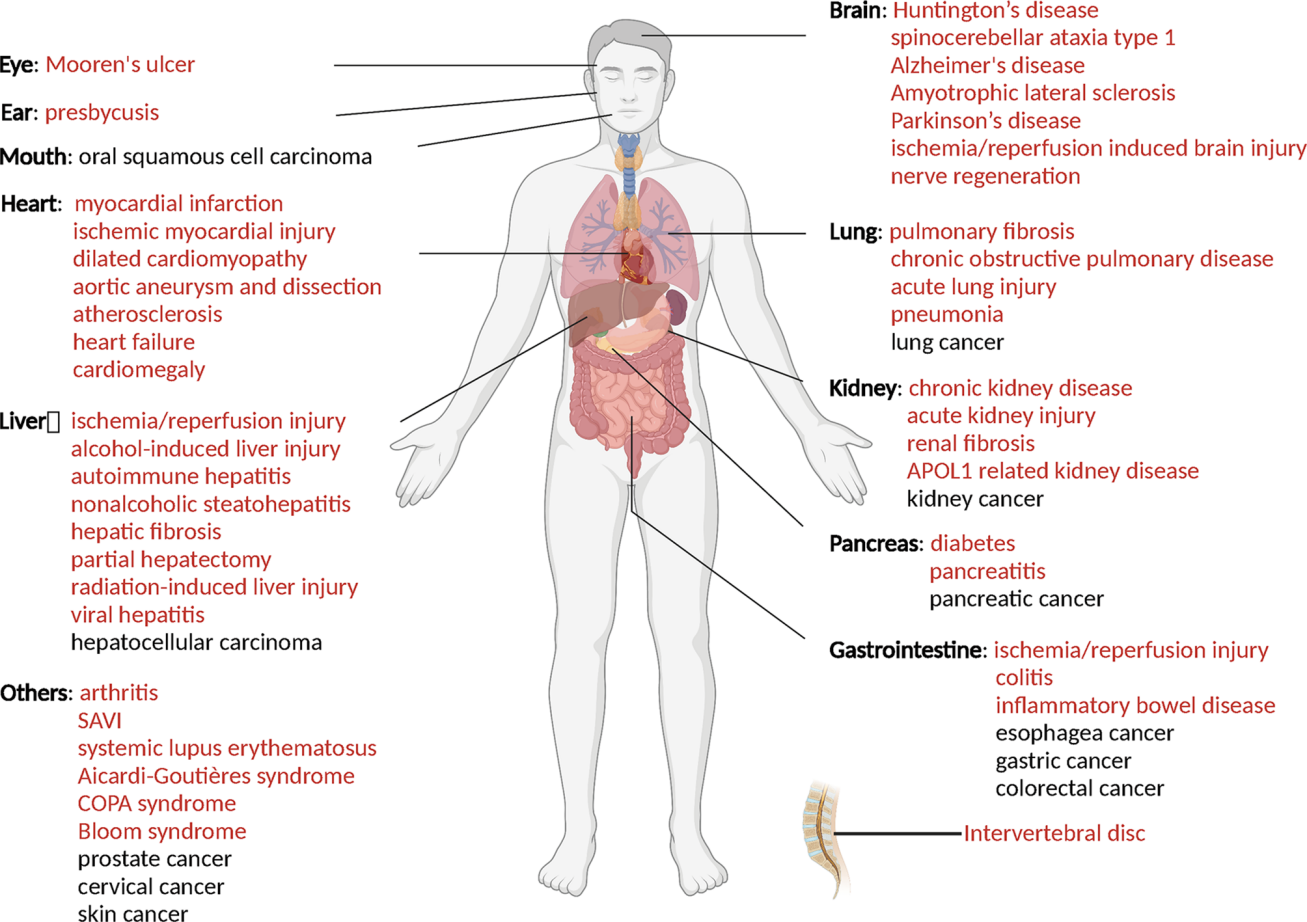
STING related diseases. Red indicates diseases caused by over-activation of STING, while black indicates disease with low-activation of STING. This figure is created with BioRender.com

As mentioned above, PTMs play critical roles in stabilization, activation and inhibition of STING, thus the immune responses could be accurately regulated in biological and pathological processes (9) (**Figure 2**). The competition of different PTMs at the same residue is important for the regulation of STING activity and can be critical factor in development of diseases, including infectious diseases (117), cancer (118), auto-inflammatory diseases (116). Therefore, targeted STING therapies have been developed on the basis of PTMs. Since phosphorylation is the classic activation PTMs form of STING, almost all of the STING-related diseases are discovered to be associated with phosphorylation of STING. Therefore, we review as follows mainly those diseases, which are covered to be related to non-phosphorylation of STING. All of STING-related diseases are presented in **Figure 3**.

### Infectious Diseases

Infection is caused by pathogens, including bacteria, virus, by which PAMPs can be produced, thus STING pathway can be activated to deal with infection (1). In most time, activation of STING can help to eliminate pathogens (4), while sometimes STING can also help pathogens survive in cells and evade from host immune surveillance (51, 119). Virus is unique, with the simple structure consisting of nucleic acid (DNA or RNA) and protein. Most studies about STING are developed in viral infection model, for example, HSV-1. On this basis, PTMs of STING are gradually discovered, especially carbonylation, ubiquitination, SUMOylation, deubiquitination, glycosylation (**Table 1**), and related strategies are implemented in virus vaccination (120). However, more studies are needed to figure out in which cases STING is beneficial for clearance of virus, and how we utilize STING in treatment of viral diseases (**Table 1**).

**Table 1:** PTMs of STING and associated study models.

Diseases	Amino acid residues	PTMs	Related molecular	Function	References
Viral infection	S366	phosphorylation	TBK1	activation	Zhong B, et al. (54)
	Y240/Y245	phosphorylation	CSK	promoting binding of STING and cGAMP, activation	Gao P, et al. (57)
	Y245	phosphorylation	EGFR	STING trafficking to endosomes, activation	Wang C, et al. (59)
	Y240	phosphorylation	SYK	activation	Wang C, et al. (60)
	S366	phosphorylation	ULK1	inhibition	Konno H, et al. (61)
	S358	dephosphorylation	PPM1A	inhibiting STING aggregation, inhibition	Li Z, et al. (62)
	Y162	dephosphorylation	SHP1	inhibition	Wang Y, et al. (63)
	–	dephosphorylation	PPP6C	inhibition	Ni G, et al. (58)
	Y245	dephosphorylation	PTPN1/2	promoting degradation of STING, inhibition	Xia T, et al. (64)
	C88/C257	carbonylation	GPX4	inhibiting palmitoylation of STING, inhibition	Jia M, et al. (111)
	K224/20/289	K63-linked ubiquitination	RNF115	promoting aggregation of STING and recruitment of TBK1, activation	Zhang ZD, et al. (67)
	K150	K63-linked ubiquitination	TRIM56	promoting STING dimerization and recruitment of TBK1, activation	Tsuchida T, et al. (68)
	–	K63-linked ubiquitination	UBXN3B	activation	Yang L, et al. (69)
	K224	K63-linked ubiquitination	MUL1	activation	Ni G, et al. (70)
	K20/150/224/236	K63-linked ubiquitination	TRIM32	promoting recruitment and activation of TBK1, activation	Zhang J, et al. (71)
	K137/150/224/236	K27-linked polyubiquitination	AMFR/INSIG1	promoting recruitment of TBK1, activation	Wang Q, et al. (74)
	K150	K48-linked ubiquitination	RNF90	enhanceing the degradation of STING	Yang B, et al. (76)
	K150	K11-linked ubiquitination	RNF26	protecting STING from RNF5-mediated degradation, activation	Qin Y, et al (77)
	K288/K337/K370	K48-linked ubiquitination	TRIM29	promoting the degradation of STING	Li Q, et al. (78) Xing J, et al (79)
	K275	K48-linked ubiquitination	TRIM30α	proteasome-dependent degradation, inhibition	Wang Y, et al (80)
	K19	K6-linked ubiquitination	TRIM13	promoting the degradation of STING	Li X, et al (82)
	K347	K48-linked deubiquitination	OTUD5	maintaining the stability of STING, activation	Guo Y, et al. (89)
	K338	SUMOylation	TRIM38	maintaining the stability of STING, activation	Hu MM, et al. (90)
	–	K63/K27-linked deubiquitination	USP21	inhibiting translocation of STING and recruitment of TBK1, inhibition	Chen Y, et al (83)
	–	K48-linked deubiquitination	USP20/USP18	maintaining the stability of STING, activation	Zhang M, et al. (88)
	–	K27-linked depolyubiquitination	USP13	preventing the recruitment of TBK1, inhibition	Sun H, et al. (84)
	K150	K48-linked ubiquitination	RNF5	promoting degradation of STING, inhibition	Zhong B, et al. (75)
	Y46/H50/P110/Y106/S108	glycosylation	sGAGs	promoting aggregation of STING, activation	Fang R, et al. (107)
	C148	oxidation	ROS	Inhibiting polymerization and activation of STING, inhibition	Tao L, et al (115)
SAVI	C88/91	palmitoylation	DHHC3/7/15	promoting clustering of STING on TGNs, activation	Mukai K, et al. (95)
	C88/91	nitro-alkylation	NO_2_-FAs	inhibiting palmitoylation of STING, inhibition	Hansen A L., et al. (99)
	C148	disulfide bond	–	promoting the dimerization of STING, activation	Ergun S L., et al. (116)
SLE	K150	K63-linked deubiquitination	MYSM1	blocking STING dimerization and aggregation and TBK1 and IRF3 recruitments, inhibition	Tian M, et al. (86)
ovarian cancer	–	K6/K11/K27/K29/K63-linked depolyubiquitination	USP35	inhibiting the binding of STING and TBK1, inhibition	Zhang J, et al. (85)

Bacterial infections are major infectious diseases worldwide, leading to many diseases, including pneumonia (121), tuberculosis (122), and sepsis (123). It is confirmed that, many gram-positive and negative bacterial (117) can release not only bacterial DNA, but also cyclic diadenosine monophosphate (c-di-AMP) and virulence factors, which can trigger cGAS-STING pathway and inflammatory responses. However, not all of STING activation is beneficial for the host. Activation of the cGAS-STING pathway can promote bacterial replication and intracellular bacterial survival after *staphylococcus aureus* and *Brucella abortus* infection (124, 125). Some important physiological or pathological processes such as blood coagulation and autophagy could also be influenced by cGAS-STING pathway after bacterial infection (117). When *Listeria monocytogenes* enters a cell, c-di-AMP can be secreted and promote activation of STING, resulting in the reduction of protective immune responses (126). Therefore, regulation and function of cGAS-STING pathway are complicated when pathogens invade. Studies about STING potential mechanism, apart from the mediator of immune responses, are still on the way.

### Cancer

To date, tumorigenesis is regarded as a process driven by inflammation (127). Previously, it is favored that the formation of tumor is related to weakened surveillance of immune system. Therefore, as an activator of immune responses, activation of STING could be potential therapy of cancer (118, 128, 129). Therapy of cGAMP or analogs into tumor-bearing mice results in substantial inhibition of tumor growth and improves the survival of the mice (130). As mentioned above, USP35 can directly deubiquitinate STING with K6/K11/K27/K29/K63-linked polyubiquitin chains and inhibit STING. Silencing USP35 potentiates cisplatin effects in ovarian cancer cells (85). Another kinase, DAPK3, is required for STING K63-linked poly-ubiquitination to activate STING pathway. However, DAPK3 loss-of-function has been discovered in several human tumor types (131), which could help tumor cells evade from host immunity and cancer immunotherapy. All of these encourage us that, cancer cells may develop anti-PTMs of STING strategy to avoid surveillance of host immune system, and targeted PTMs of STING could be potential therapy for cancer, especially those STING-sensitive cancers (**Table 1**).

### Autoimmune and Inflammatory Disease

Mutation or abnormal activation of STING can lead to varieties of auto-immune and auto-inflammatory diseases, including systemic lupus erythematosus (SLE) and STING-associated vasculopathy with onset in infancy (SAVI). SLE is a typical auto-immune disease, in which pathogenic auto-antibodies are produced, resulting in excessive inflammation and severe tissue damage (132). It remains controversial whether STING plays necessary role in SLE (133). However, MYSM1 has been discovered as a suppressor of SLE, which actually triggers K63-linked deubiquitination of STING and inhibits STING pathway. Increased MYSM1 can decrease type I IFN, IL-6, and other inflammatory cytokines in SLE mice (86). This indicates us that targeted ubiquitination of STING therapy could help improve auto-immune and inflammatory diseases.

SAVI is an auto-inflammatory disease with the gain-of-function mutations of STING (V147L, V147M, N154S, V155M, C206Y, R281Q, R284G), characterized by early onset systemic inflammation, vasculopathy and interstitial lung disease (ILD) (134, 135). Studies have discovered that, mutants of STING in SAVI are accompanied by enhanced STING translocation, IRF3 phosphorylation, and IFN-β activity. Blocking C148-mediated disulfide bond can alleviate inflammatory responses in SAVI-modeled cells (116). In addition, treatment of NO_2_-FAs, which can alkylate STING and inhibit palmitoylation, can decrease production of type I IFN in fibroblasts cultured from SAVI patients. Also, 2-BP can decrease type I IFN in SAVI-mutant HEK293T cells (99). All of these indicate that inhibition of palmitoylation and conformational change of STING, or promotion of nitro-alkylation could be potential target to treat SAVI, which reveal the importance of PTMs of STING in diseases.

### PTMs Targeted Therapy

Considering the critical role of STING signaling pathway in inflammation and diseases, targeting STING may lead to novel therapeutics (27, 136). Many strategies are developed, including: production of STING, activation of STING, translocation and oligomerization of STING, degradation of STING and downstream signaling (137). Targeted these strategies develop agonists and inhibitors, some of which are related to PTMs (**Table 2**).

**Table 2:** Structure and function of STING agonists and inhibitors.

Inhibitors					
Compound type	Compound name	Structural formula	Function	Disease	References
vermiculine	LH519	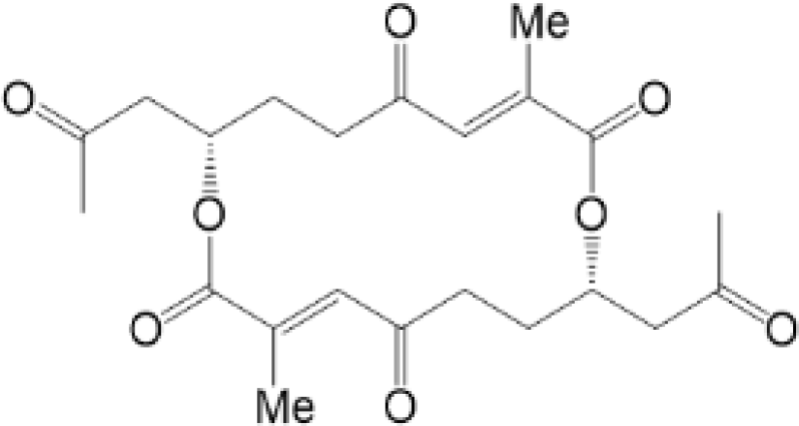	blocking phosphorylation of STING	–	Liu H, et al (155)
	LH531	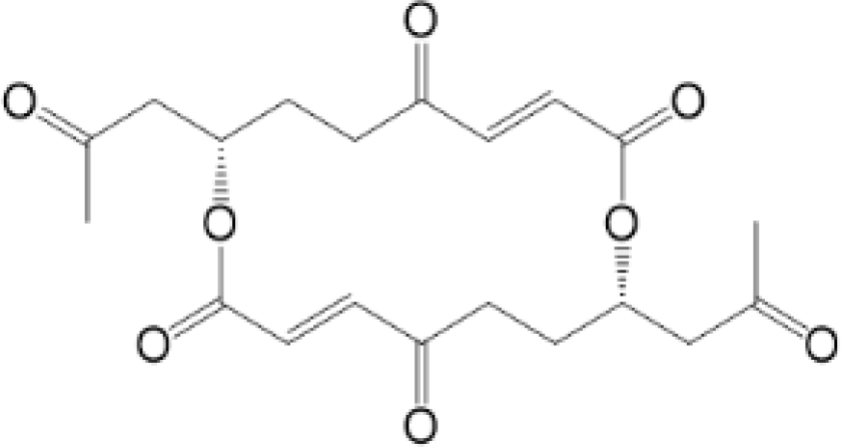	blocking phosphorylation of STING	–	
nitrofuran derivatives	C176	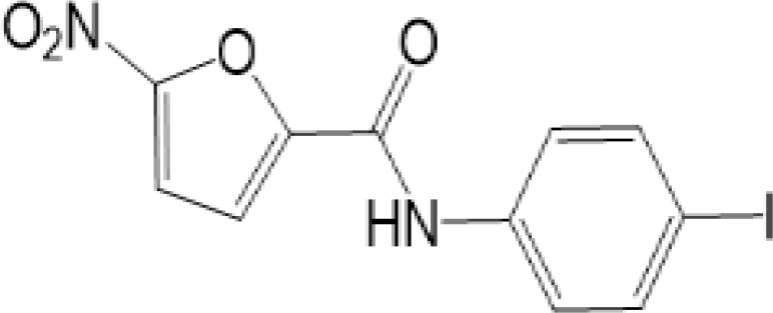	decreasing expression of STING	acute lung injury	Wu B, et al (156)
			inhibiting the palmitoylation of STING	Trex1-/-	Haag, S. M. et al (96)
	C178 H151	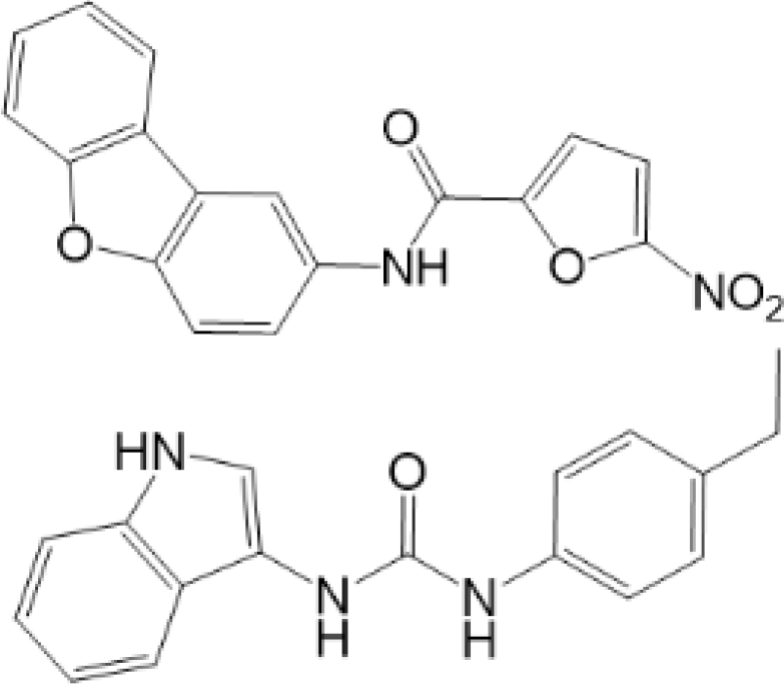	inhibiting the palmitoylation of STING inhibiting the palmitoylation of STING	Trex1-/- Trex1-/-	Haag, S. M. et al (96) Haag, S. M. et al (96)
NO2-FA	9-NO2-OA	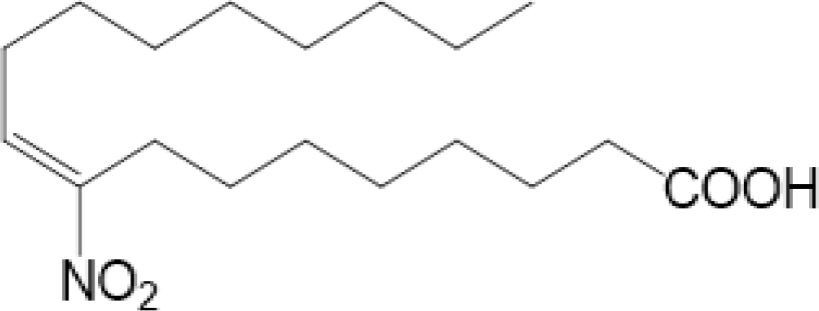	promoting nitro-alkylation and inhibiting the palmitoylation of STING	SAVI, viral infection	Hansen, A. L.et al (92), Hansen, A. L.et al (99)
	10-NO2-OA NO2-cLA	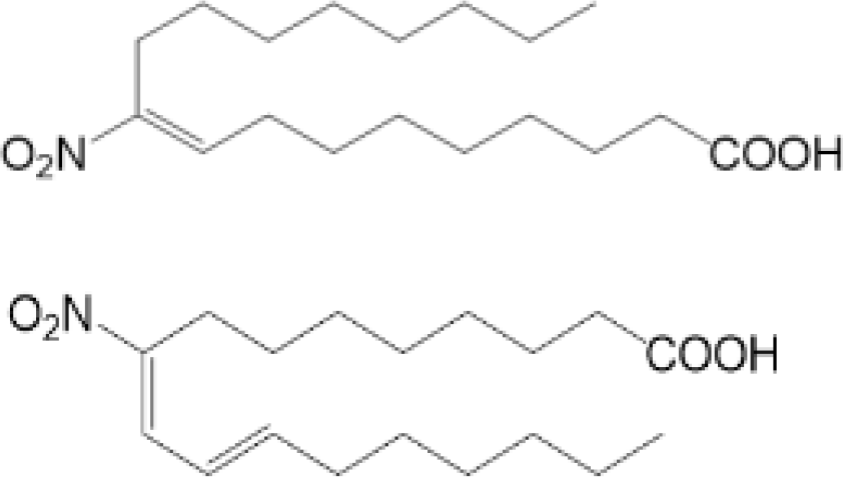			
	2-BP 4-HNE	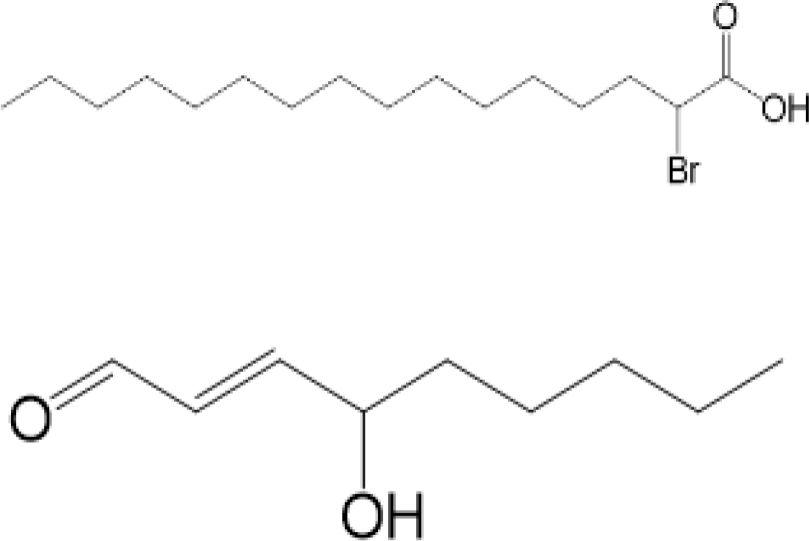	inhibiting the palmitoylation of STING inducing STING carbonylation and inhibiting the palmitoylation of STING	viral infection viral infection	Mukai K, et al (95) Jia M, et al (111)
cyclopeptide	astin C	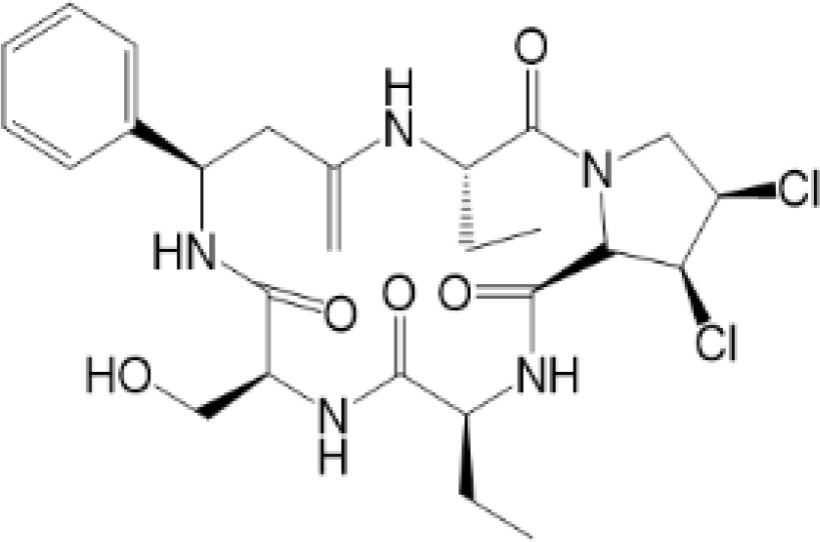	blocking the recruitment of IRF3 to STING	viral infection	Li S, et al (157)
Benzodioxane Variants	compound18	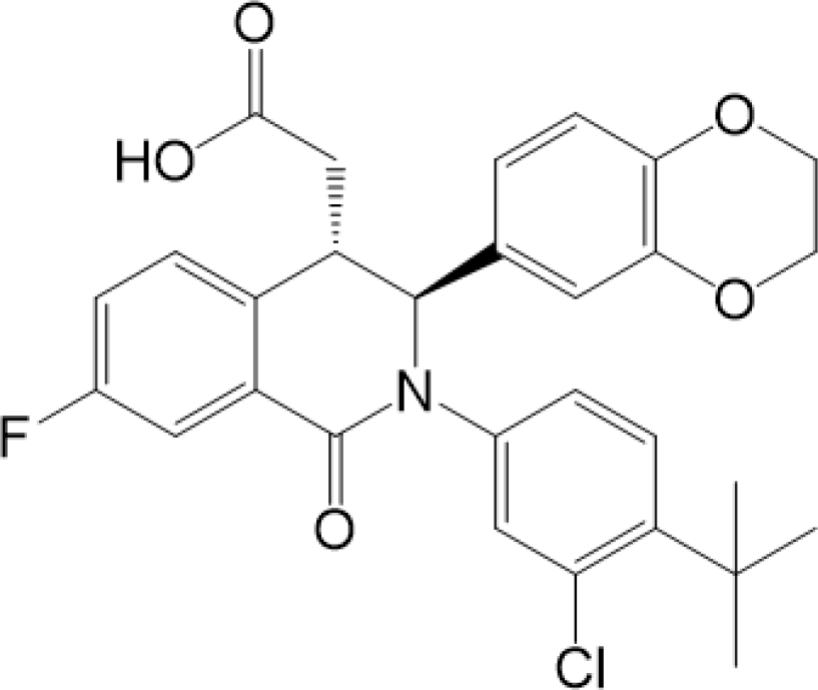	inhibiting binding of 2'3'-cGAMP and STING	–	Siu T, et al (145)
compounds containing a benzene-1-sulfonamido-3-amide group	SN-011	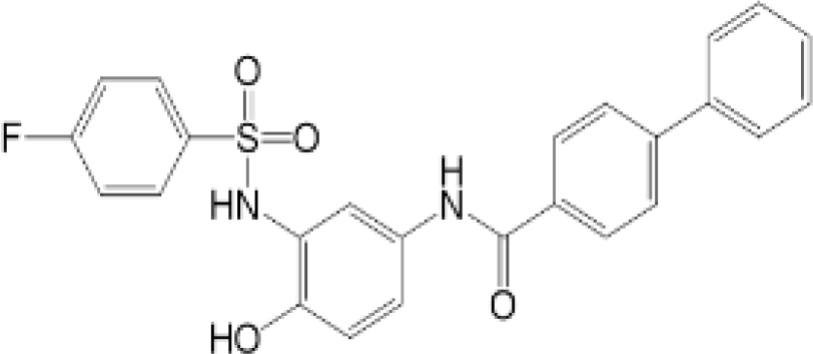	blocking CBD of STING and inhibiting oligomerization and phosphorylation of STING	Trex1-/-	Hong Z, et al (146)
ester alkaloids	homoharringtonine(HHT)	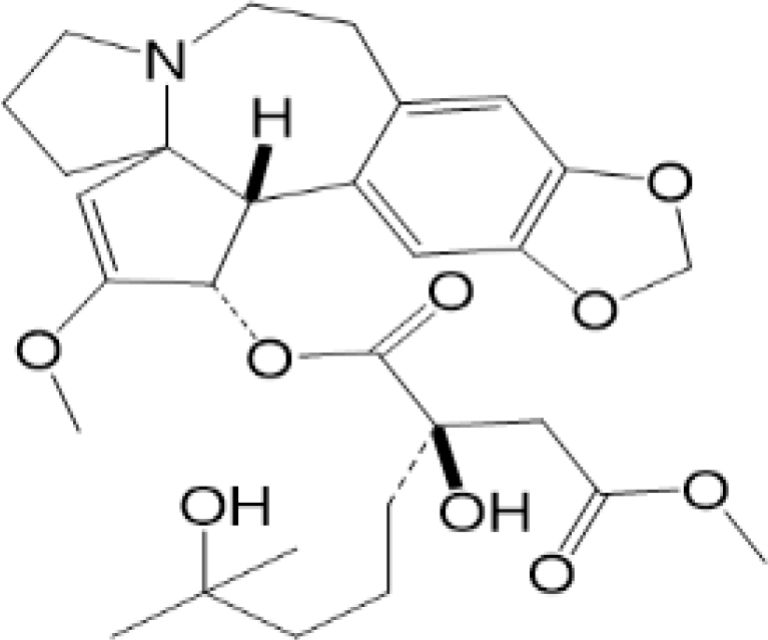	inhibiting interaction of STING and TBK1	–	Park G, et al (158)
flavonol	Kaempferol (KPF)	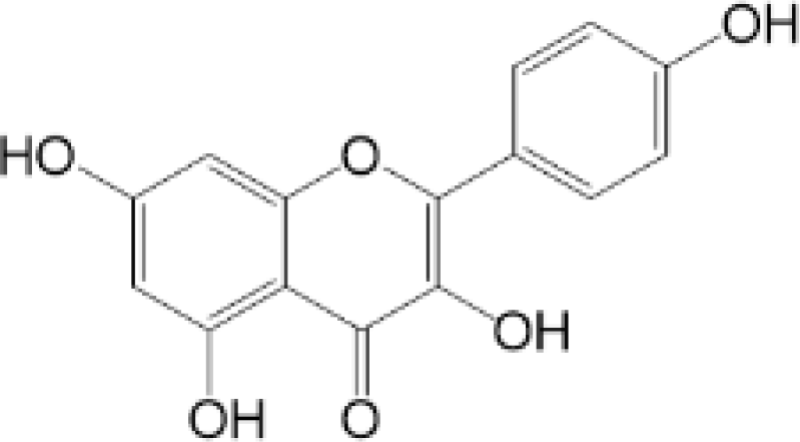	blocking phosphorylation of STING	cisplatin-induced cardiac injury	Qi Y, et al (159)
**Agonists**					
amidobenzimidazole	diABZI-4	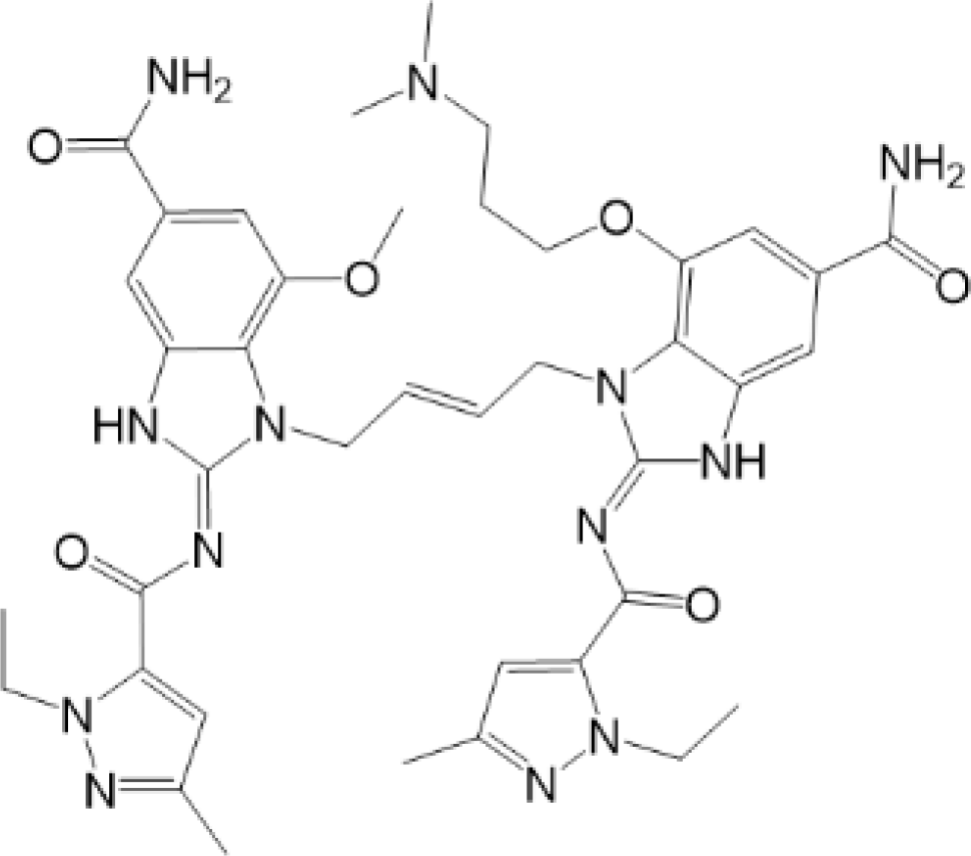	inducing oligomerization of STING	viral infection	Humphries F, et al (160)
	compound 2	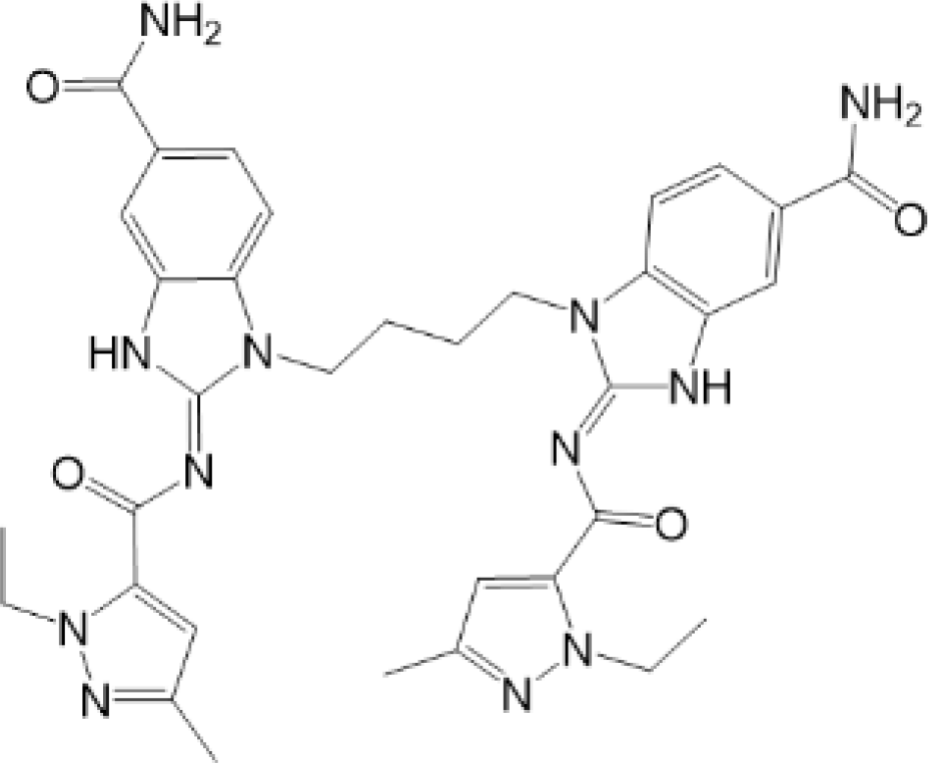	inducing phosphorylation of STING	colorectal tumours	Ramanjulu, J. M, et al (161)
	diABZI	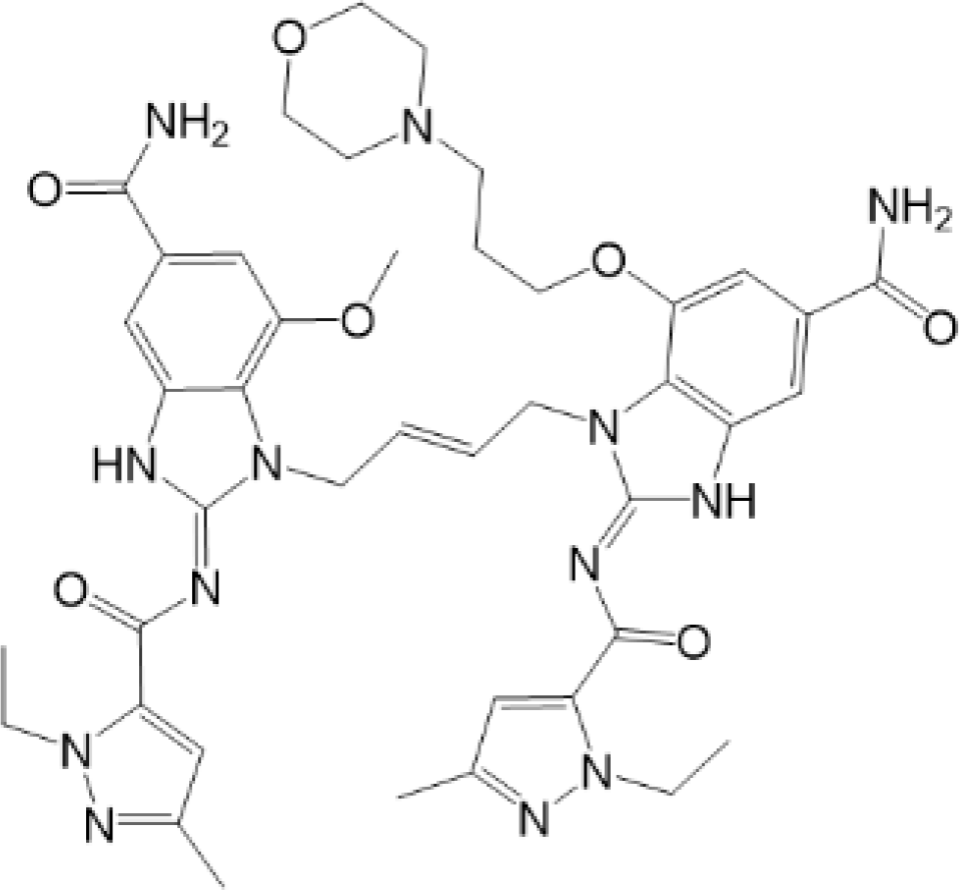	inducing phosphorylation of STING	viral infection	Zhou Z, et al (141)
	24b	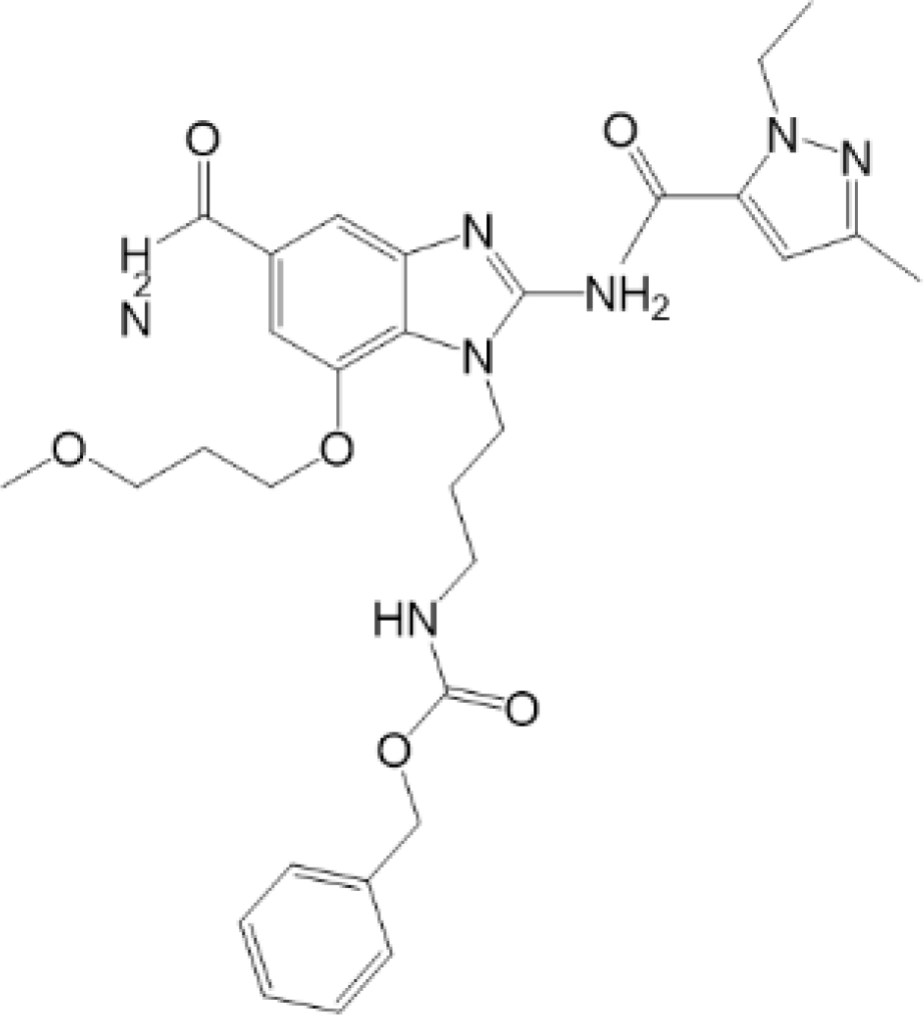	inducing phosphorylation of STING	colorectal tumours	Xi Q, et al (162)
3,4-dihydroquinazolin-2(1H)-one cyclic urea	compound92	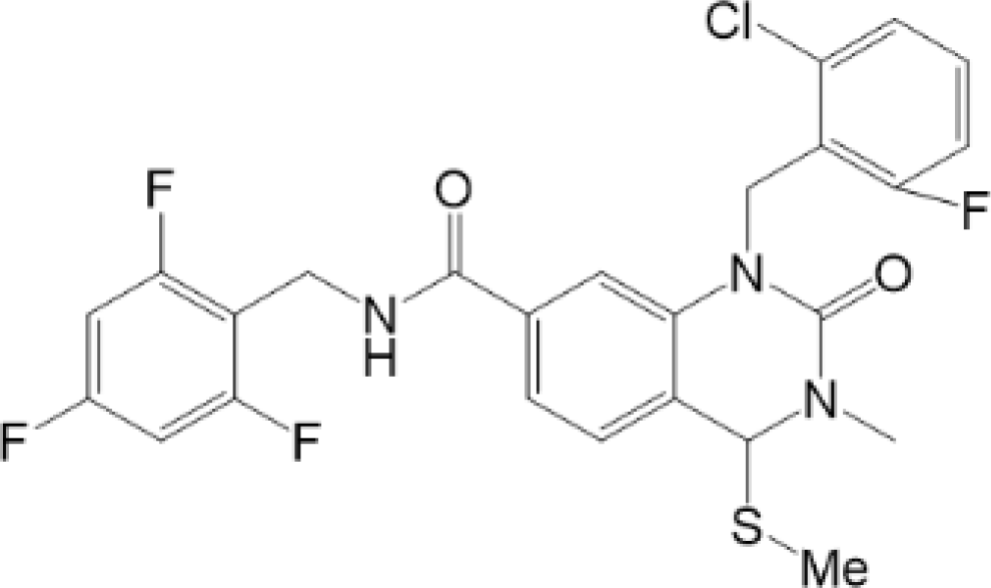	inducing phosphorylation of STING	–	Basu S, et al (163)
benzothiazinone	compound53	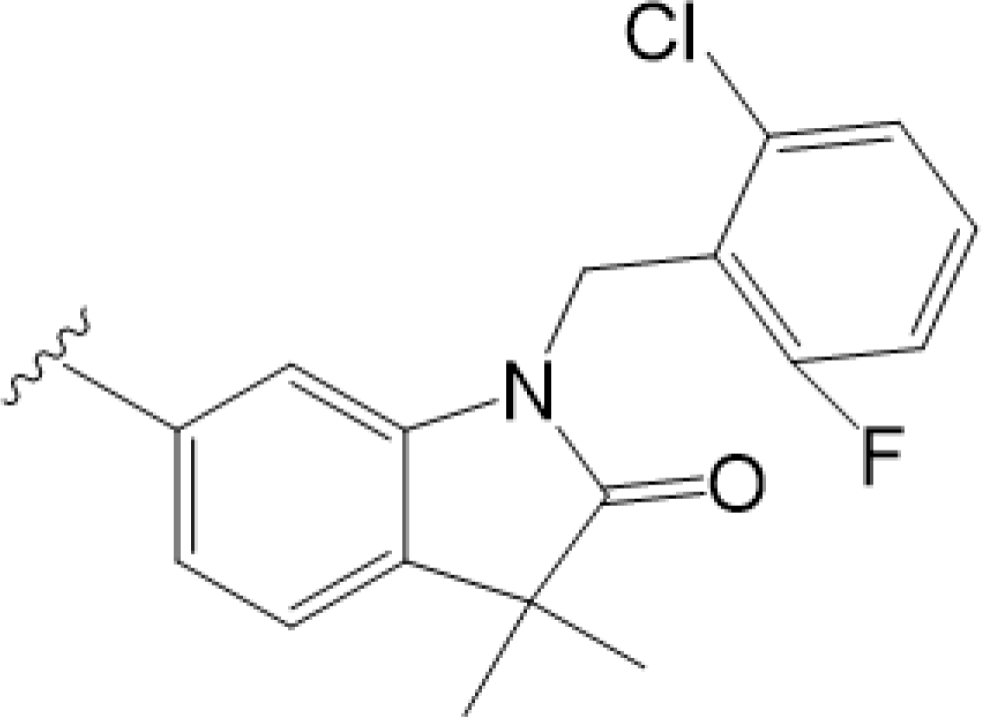	inducing phosphorylation of STING	–	Pryde, D.C., et al (142)
2-(cyclohexylsulfonyl)-N,N-dimethyl-4-tosylthiazol-5-amine	M04	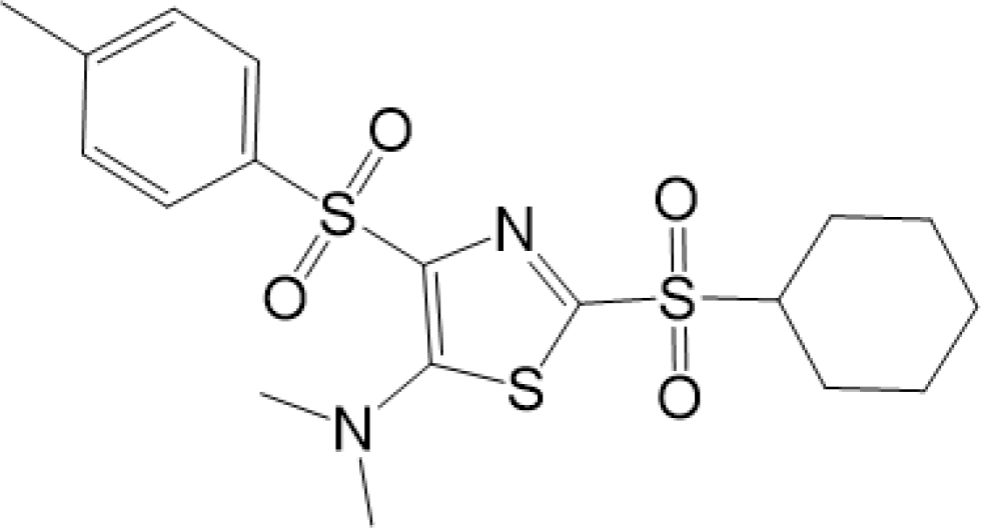	inducing phosphorylation and trafficking of STING	viral infection	Abraham, J, et al (143)
triazoloquinoxaline	1a	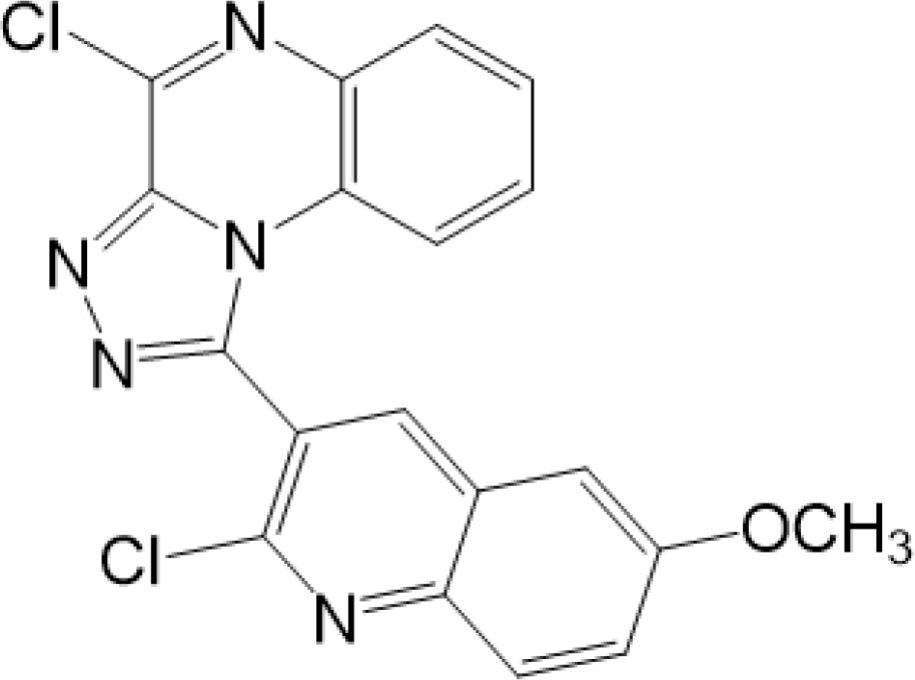	inducing phosphorylation of STING	–	Hou H, et al (164)
–	SINCRO	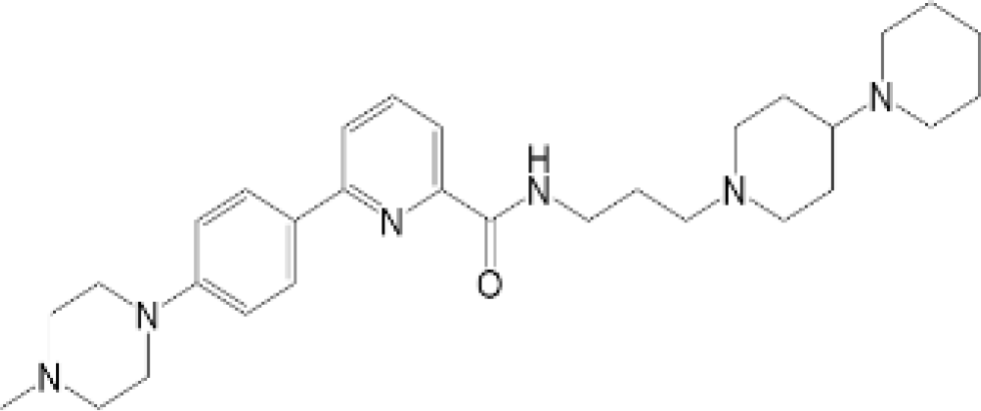	promoting activation of STING	melanoma	Kimura, Y, et al (165)
1H-benzimidazole-4-carboxamide derivatives	CHX710	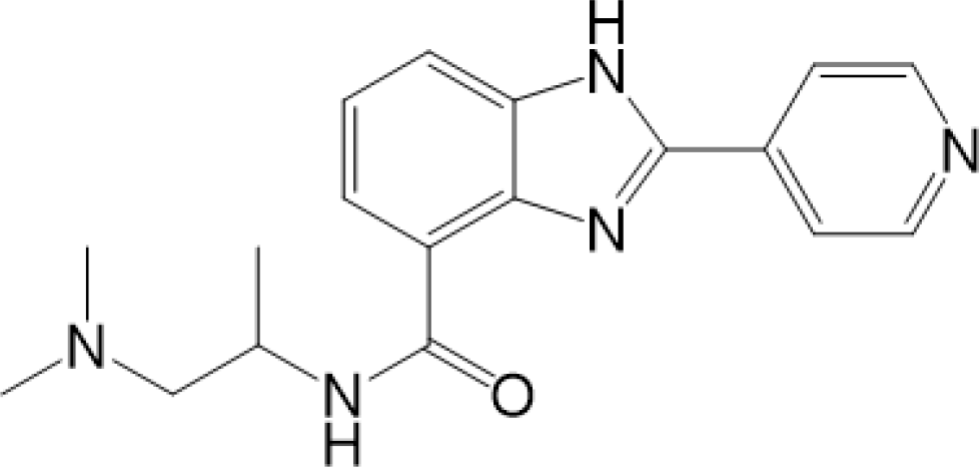	activation of STING	–	Khiar, S, et al (166)
sulfonylureas	DW2282	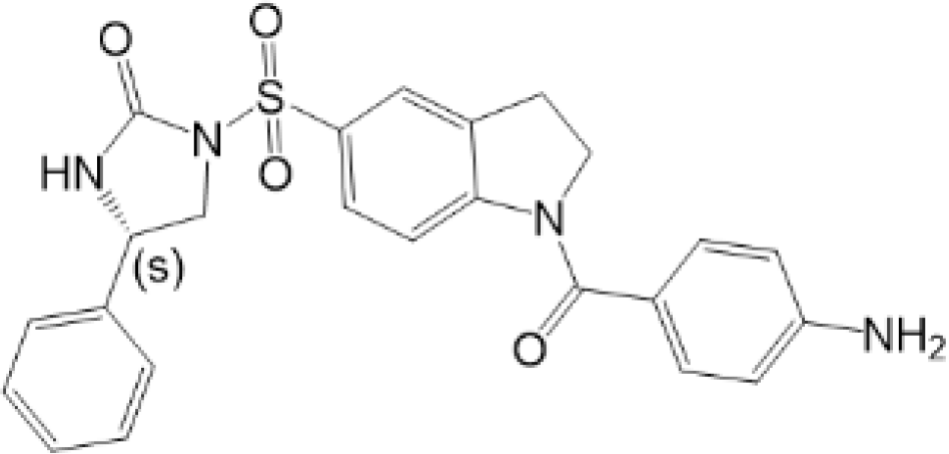	inducing phosphorylation of STING	colorectal tumours	Jung, H. R., et al (140)
	KAS-08	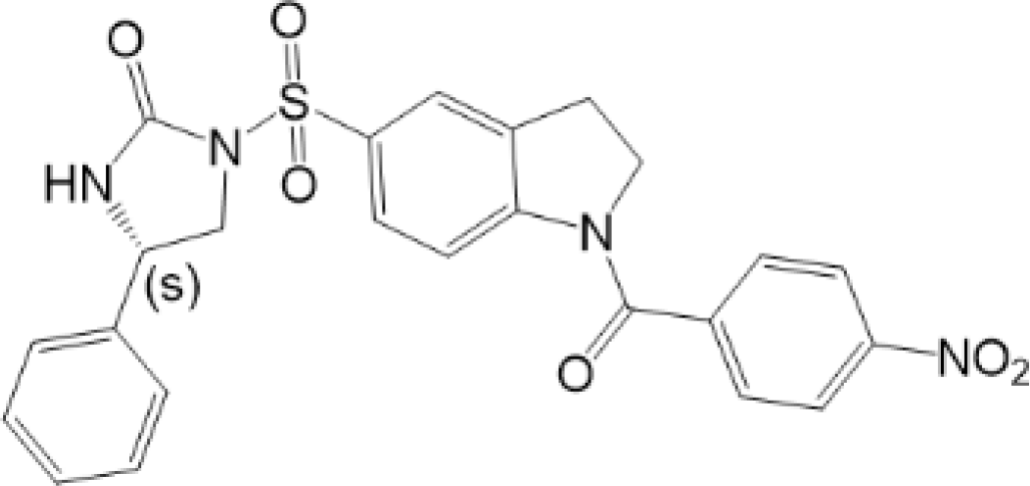	inducing phosphorylation of STING
isoquinoline alkaloid	Cepharanthine (CEP)	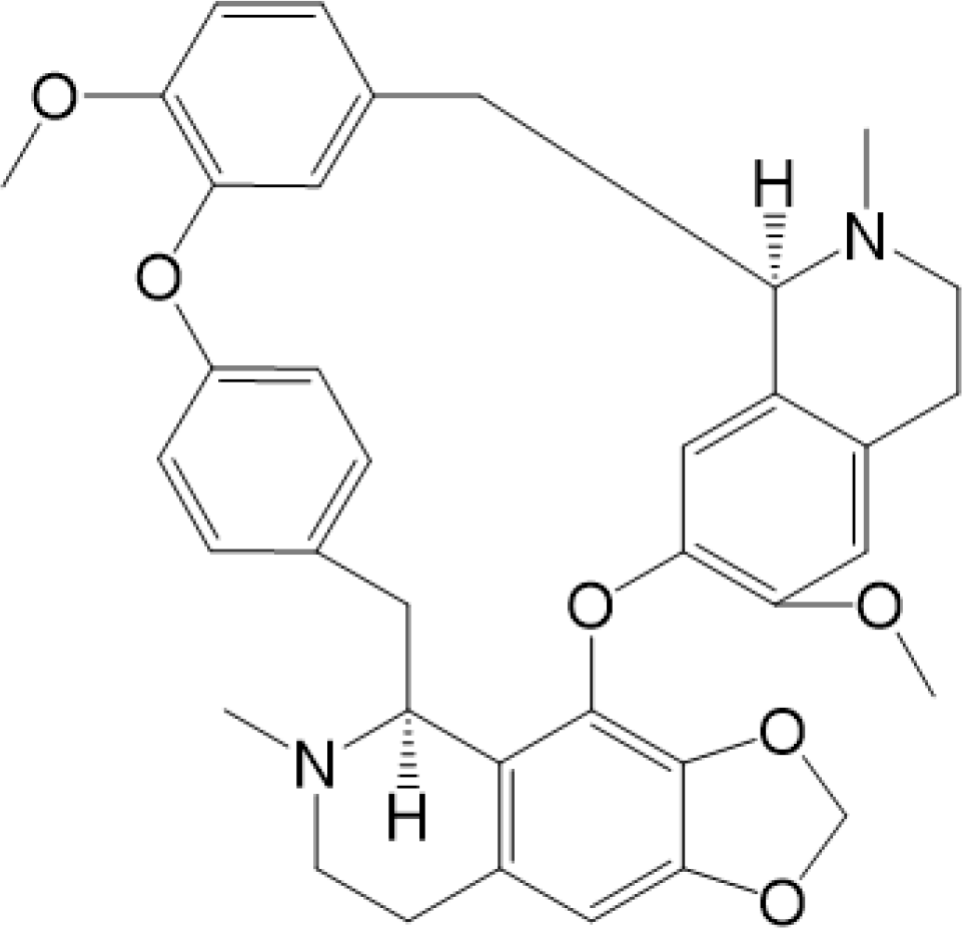	inducing phosphorylation of STING	viral infection	Liu Y, et al (139)
acridone	compound 12b	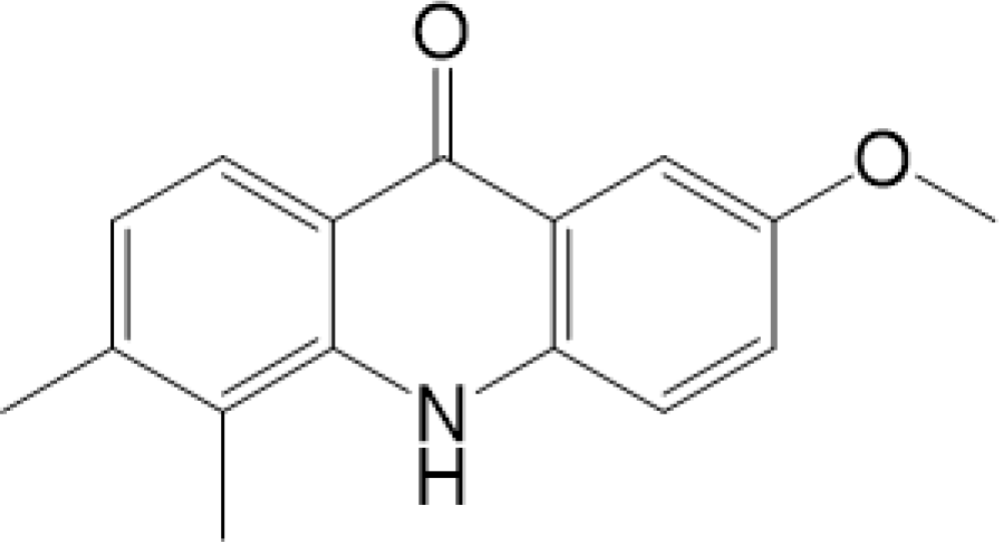	activation	–	Hou S, et al (167)
–	compound 22	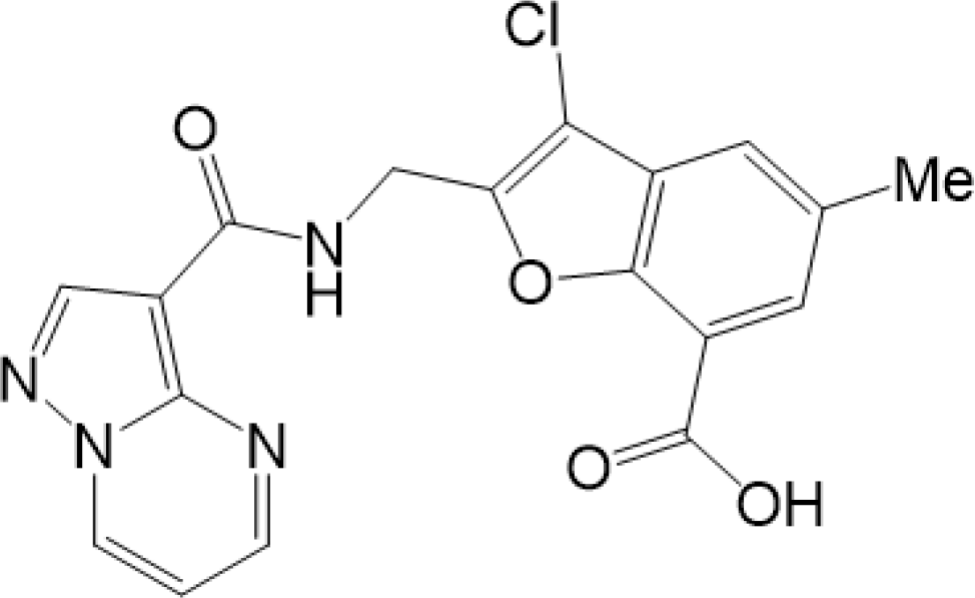	activation	colorectal tumours	Cherney, E. C., et al (168)
carboxamide	BNBC	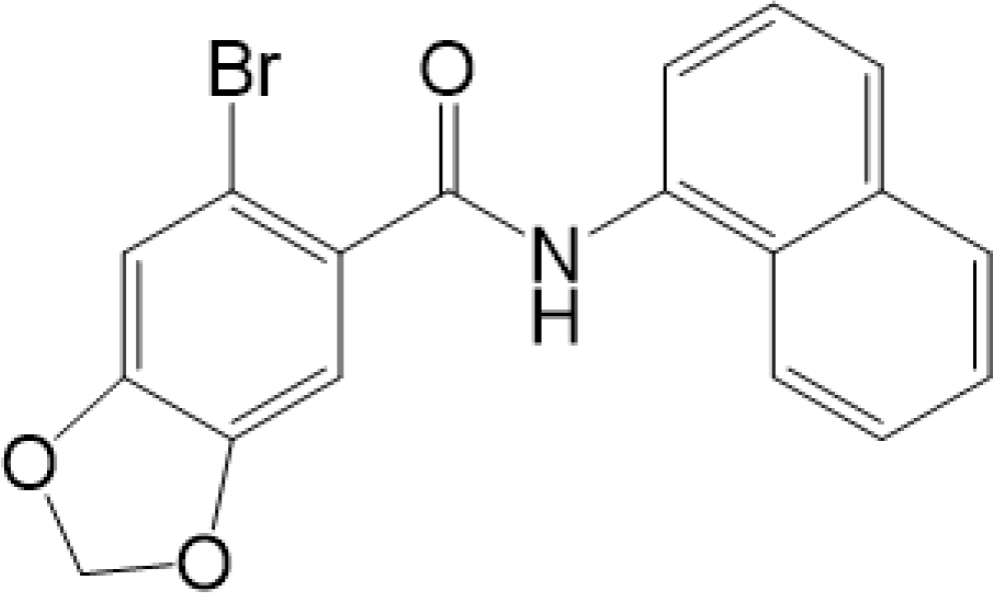	inducing the peri-nuclear translocation	viral infection	Zhang X, et al (169)

Agonists of STING have been developed for a long time. The most efficient agents are CDNs, which are fit to LBD of STING, resulting in rapid activation of STING pathway (137). Later, more agonists are reported and some of them have been applied into clinical trials to help with symptoms or enhance treatments, especially cancer (137). Novel STING agonist strategies include: bacterial vectors, CDNs, non-CDNs, nano vaccines, antibody-drug conjugate, exo-STING, etc. (160) (**Table 3**). In addition, other potential therapeutic agents have been explored in experiments *in vivo* or *in vitro*, such as CEP (156), KAS-08 (155), DW2282 (155), etc. These agents are mostly tightly related to phosphorylation of STING, which is considered as the marker of STING activation. It has been confirmed that diABZI (147), compound 53 (150), M04 (151), etc, regulate directly phosphorylation of STING (**Table 2**). While bisphenol A (BPA) can up-regulate ZDHHC1, a palmitoylase, indicating the underlying therapy of palmitoylation in regulation of STING (161). Therefore, PTMs play critical role in strategies of agonists. All of these agonists activate immune responses through cGAS-STING pathway, thus play a critical role in anti-cancer therapy.

**Table 3:** STING agonists in clinical development.

AGENT		PHASE	TYPE OF CANCER	TIME	CLINICAL TRIAL NCT CODE
E-7766	single agent	phase I	advanced solid tumors or lymphomas, melanoma, head and neck squamous cell carcinoma (HNSCC), breast cancer, colorectal cancer, and/or other tumors including lymphomas	2020.3-2022.12	NCT04144140
exoSTING(CDK-002)	single agent	phase II	advanced/metastatic, recurrent, injectable solid tumors	2020.9-2022.12	NCT04592484
IMSA-101	single agent or+Immune checkpoint inhibitor (ICI)/Immuno-oncology (IO) therapy	phase I/II	Advanced Treatment-Refractory Malignancies	2019.9-2023.2	NCT04020185
ADU-S100	Single agent or + Ipilimumab	phase I	Advanced/Metastatic Solid Tumors or Lymphomas	2016.4-2020.8	NCT02675439
	+Pembrolizumab	phase II	Head and Neck Cancer	2019.8-2021.6	NCT03937141
	+PDR001	phase Ib	Advanced/Metastatic Solid Tumors or Lymphomas	2017.9-2020.12	NCT03172936
MK-1454	Single agent or + Pembrolizumab	phase I	Advanced/Metastatic Solid Tumors or Lymphomas	2017.2-2022.10	NCT03010176
	+Pembrolizumab	phase II	Metastatic or Unresectable, Recurrent Head and Neck Squamous Cell Carcinoma	2020.3-2023.4	NCT04220866
TAK-676	+Radiation+Pembrolizumab	phase I	Non-small-cell Lung Cancer, Triple-negative Breast Cancer, or Squamous-cell Carcinoma of the Head and Neck	2021.9-2024.1	NCT04879849
	Single agent or + Pembrolizumab	phase I	Advanced or Metastatic Solid Tumors	2020.7-2023.3	NCT04420884
SB-11285	Single agent or + Atezolizumab	phase I	Advanced Solid Tumors	2019.9-2022.5	NCT04096638
SYN-STING	Single agent or + Atezolizumab	phase I	Advanced/Metastatic Solid Tumors and Lymphoma	2019.11-2023.6	NCT04167137
GSK-3745417	Single agent or + Pembrolizumab	phase I	refractory/relapsed solid tumors	2019.3-2025.1	NCT03843359
BI-1387446	single agent or +BI 754091	phase I	Solid Tumors	2020.3-2025.1	NCT04147234
SNX-281	Single agent or + Pembrolizumab	phase I	Advanced Solid Tumors and Lymphoma	2020.11-2024.3	NCT04609579
BMS-986301	Single agent or + Nivolumab/Ipilimumab	phase I	Advanced Solid Cancers	2019.3-2024.7	NCT03956680
TAK-500	Single Agent or + Pembrolizumab	phase I	Select Locally Advanced or Metastatic Solid Tumors	2022.1-2025.4	NCT05070247
MK-2118	+ Pembrolizumab	phase I	Advanced/Metastatic Solid Tumors or Lymphomas	2017.9-2022.6	NCT03249792

As for inhibitors, strategies have also been developed to inhibit STING pathway: decreasing the expression of STING, blocking binding of 2’3’-cGAMP and LBD of STING, inhibiting phosphorylation, blocking traffic of STING, etc (27). Compound 18 is discovered to inhibit binding of 2’3’-cGAMP and STING, leading to inhibition of STING pathway (141). Other inhibitors are mainly associated with PTMs of STING. C176/C178/H151 have been discovered to inhibit palmitoylation of STING (96), while NO_2_-FAs result in nitro-alkylation to competitively inhibit palmitoylation (99). SN-011 can block phosphorylation of STING to decrease production of type I-IFN and inflammatory cytokines (142). Therefore, PTMs of STING can be important therapeutic target to regulate STING in inflammatory diseases. Targeted PTMs could be potential efficient strategy to improve related diseases.

In a word, many therapeutic targets related to STING are based on phosphorylation, palmitoylation (92), alkylation (99). There are few studies on agonists or inhibitors related to other PTMs, for example, ubiquitination, glycosylation, sumoylation, oxidation, carbonylation, which are also critical in regulation of STING. There have been developed many reviews about targeted therapy of ubiquitination (162–164), glycosylation (165), sumoylation (166), oxidation (167). More studies could focus on the role of these agonists and inhibitors in regulating STING pathway, proposing more comprehensive therapeutic strategies to improve STING-related diseases.

## Summary and Future Perspective

Innate immune response is an important defender to deal with endogenous and exogenous abnormal situation in cells. As mentioned above, STING pathway participates importantly in regulation of immune responses (4). Since the discovery of the cGAS-STING pathway, a series of biochemical, structural and genetic studies have been conducted and related mechanisms have been established. It is confirmed that, PTMs of STING are important in regulation of STING pathway (9). Many types of PTMs participate in regulation of activation or degradation of STING (12), including phosphorylation and dephosphorylation, palmitoylation, nitro-alkylation, glycosylation, ubiquitylation and deubiquitylation, SUMOylation, carbonylation, oxidation and so on, so that immune responses induced by STING could be activated or inhibited efficiently.

It is inspiring that the same amino acid residues of STING could be modified by different groups, resulting in distinct, even contrary consequences. For example, C88 could be palmitoylated to activate immune responses (92), while ROS and NO_2_-FA could result in carbonylation and alkylation of STING to inhibit production of type I IFNs (99, 111). Add or remove phosphorylated groups on Y245 and S366 can also function differently, which indicates the importance of PTMs to precisely regulate STING (56, 57, 61, 64).

Due to important role played by STING in immune responses and inflammation, the critical mechanism of PTMs of STING is deserved to explore and transform into potential therapeutic target. Since then, targeted therapy of STING has been highlighted in treatment of many diseases (27, 137). Most agonists have been explored to improve prognosis of cancer (168). Among them, the most common mechanism is to phosphorylate STING to activate immune responses. By contrast, nitrofuran derivatives, astin C and others are inhibitors of STING (96, 169), which could potentially help with auto-immune diseases and hyper-activation of inflammation. However, the potential mechanisms of those molecular agents are not clearly clarified, which limits wider use of these drugs. In a word, due to complexity of immunity regulation, much remains to be learned about the regulation of STING and details of PTMs in STING pathway. Future therapeutic strategies could focus on optimizing PTMs on STING function in the right disease at the optimal time.

## Author Contributions

JK developed and wrote this review. JW and QL revised it critically for important intellectual content. XW, YZ, and JR provide approval for publication of the content. All authors contributed to the article and approved the submitted version.

## Funding

The work was supported by National Natural Science Foundation of China (81772052, 82072149, 82072223, and 82100597), the Natural Science Foundation of Jiangsu Province (BK20201116, BK20210039), the program B for Outstanding PhD candidate of Nanjing University.

## Conflict of Interest

The authors declare that the research was conducted in the absence of any commercial or financial relationships that could be construed as a potential conflict of interest.

## Publisher’s Note

All claims expressed in this article are solely those of the authors and do not necessarily represent those of their affiliated organizations, or those of the publisher, the editors and the reviewers. Any product that may be evaluated in this article, or claim that may be made by its manufacturer, is not guaranteed or endorsed by the publisher.
